# Late Identification of a Stealthy Aorto-Pulmonary Window: A Case Report

**DOI:** 10.7759/cureus.42524

**Published:** 2023-07-27

**Authors:** Najlaa Belharty, Meriem Elharrak, Ghizlan Ziani, Tanae El Ghali, Mohamed Cherti

**Affiliations:** 1 Department of Cardiology B, Ibn Sina Hospital, Mohammed V University, Rabat, MAR

**Keywords:** pulmonary trunk, ascending aorta, case report, echocardiography, aortopulmonary septal defect, aortopulmonary window

## Abstract

The term "aortopulmonary window" (APW), often referred to as "aortopulmonary septal defect," refers to a rare congenital medical disorder where there is an improper direct link between the main pulmonary artery and the ascending aorta. It can be combined with other cardiac congenital conditions or be an isolated lesion.

Herein, we report the incidental discovery of a minor, restrictive aortopulmonary septal defect in a 60-year-old male who denied having any clinical symptoms. Incidentally detected APW in adulthood is uncommon and, hence, can be readily overlooked, a fortiori, in asymptomatic patients.

## Introduction

Aortopulmonary window (APW) refers to an improper connection between the ascending aorta and the main pulmonary artery (MPA) with integral and distinct aortic and pulmonary valves, which results in a left-to-right shunt [1-2]. Adult APW presentation is infrequent and rarely reported in the literature; hence, there is limited data on the management of APW beyond early childhood [2]. We report a rare case of a very late, incidental discovery of an aortopulmonary window with no clinical symptoms and no associated congenital abnormalities.

## Case presentation

A 60-year-old Moroccan male with a history of well-controlled hypertension presented to our cardiology department for a routine echocardiography study.

The patient denied any chest pain, tiredness, or dyspnea. Physical inspection showed a normal blood pressure of 130/60 mmHg, a normal heart rate of 80 bpm, a respiratory rate of 16 cpm, a normal oxygen saturation of 97%, and no heart murmur.

**Figure 1:**
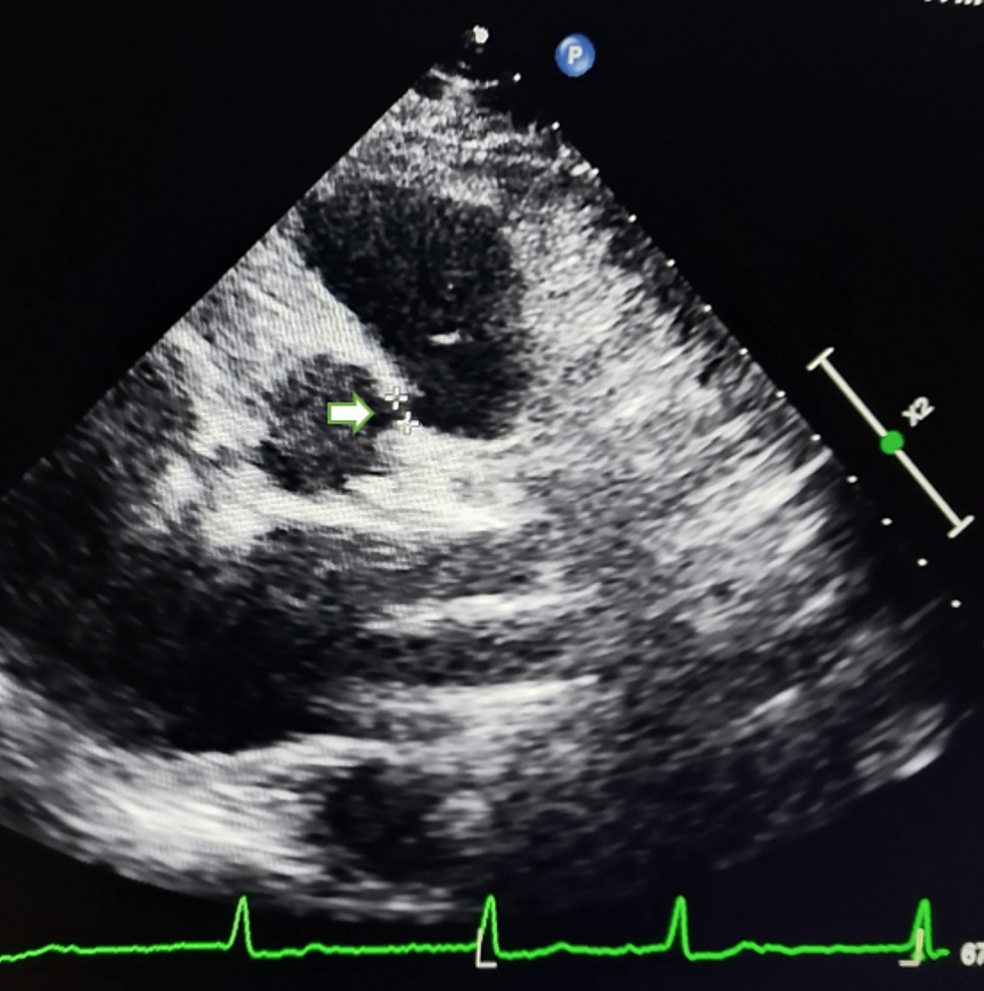
Bi-dimensional echocardiography, on short axis view, demonstrating the small size of the aortopulmonary window (white arrow), above the pulmonary valve

**Figure 2:**
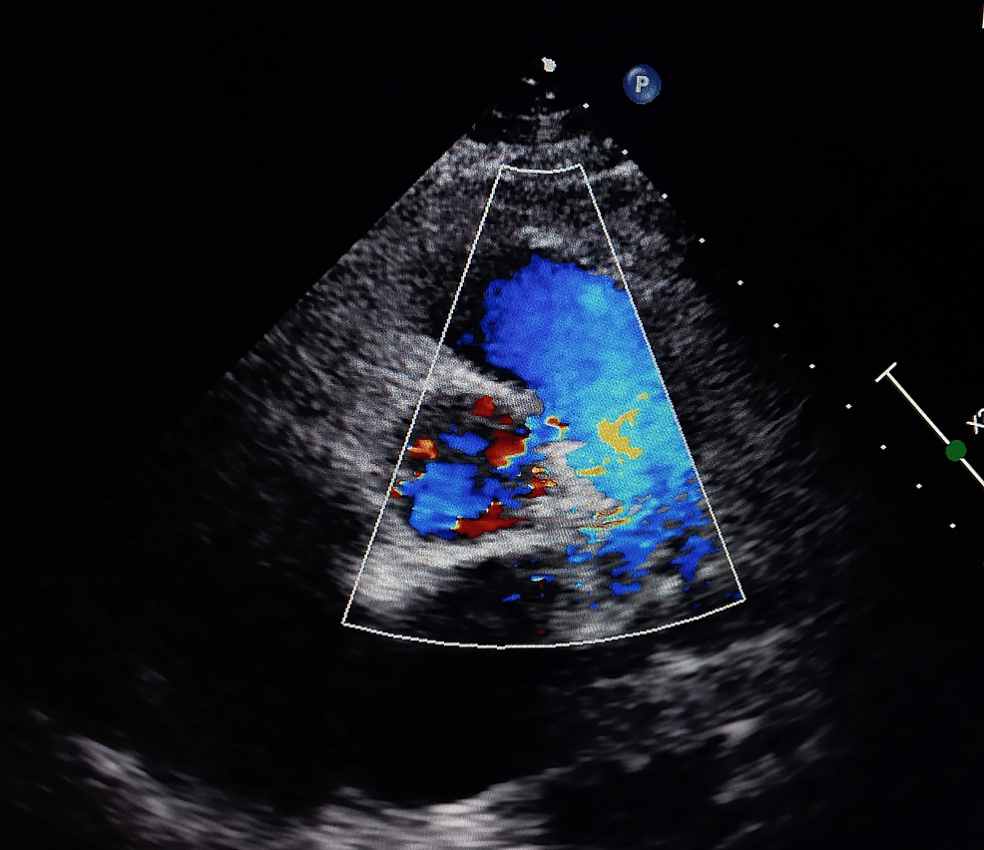
Color Doppler echocardiographic view showing flow across the aortopulmonary septal defect

On the parasternal short axis view, echocardiography revealed modest connectivity between the ascending aorta and the right side of the pulmonary trunk of about 4 mm in width (Figure 1), with a left-to-right shunt on color Doppler (Figure 2).

**Figure 3:**
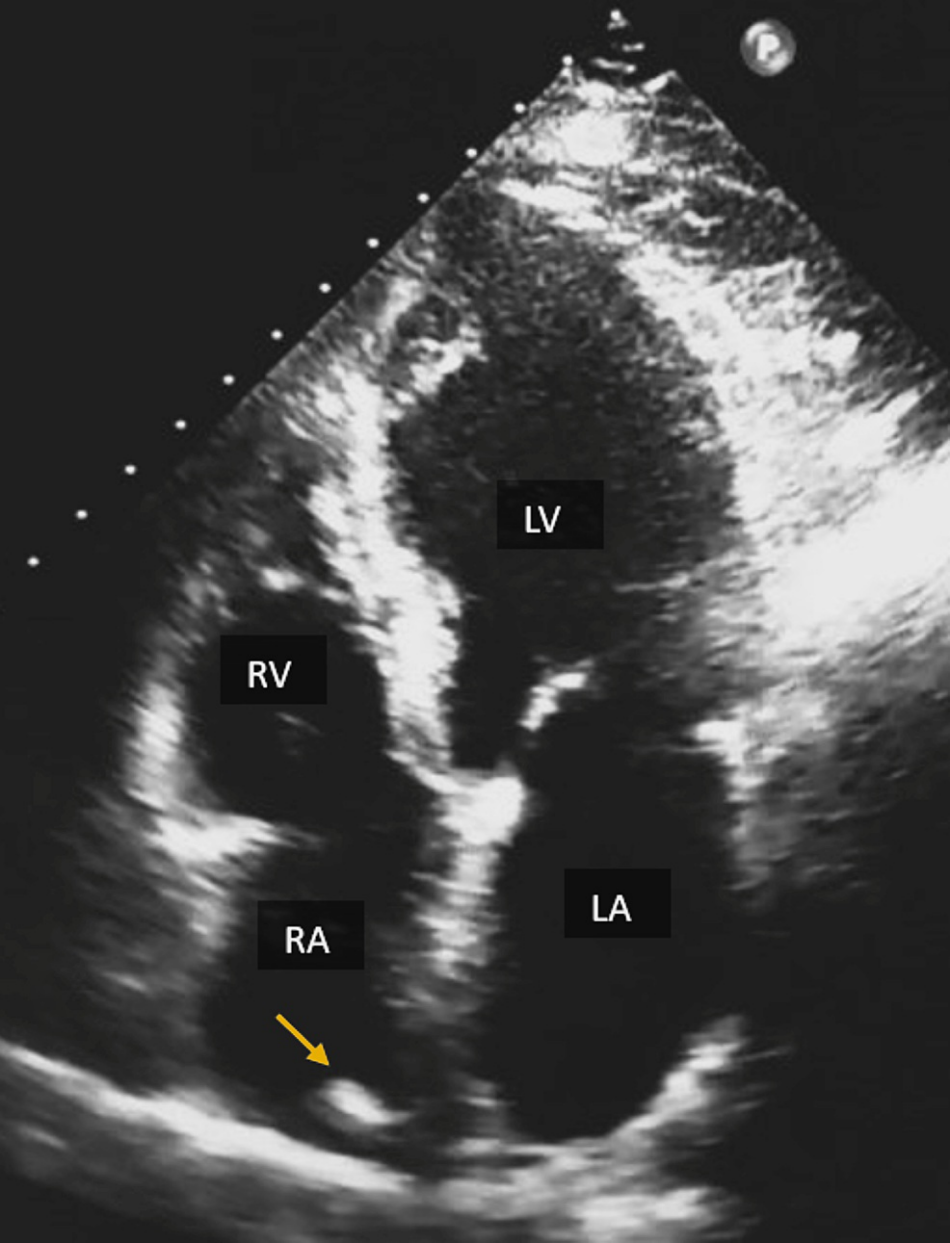
The apical four-chamber view visualizes mildly dilated left (LV) and right ventricles (RV) with a ventricular septal bulge and mild dilatation of the left atrium (LA). Transthoracic echocardiography also reveals the presence of the crista terminalis, which is a normal variant in the right atrium (RA) (arrow).

The left atrium, right, and left ventricles, as well as the pulmonary trunk, were all mildly dilated. Mild regurgitation of the mitral and tricuspid valves was also detected (Figure 3).

Although there was a little reduction in left chamber function, no congenital defects were found to be related. Additionally, there was echocardiographic evidence of an intermediate probability of pulmonary hypertension (PH) and preserved right ventricular function.

Sadly, the patient was lost for follow-up and hasn’t undergone any further investigations.

## Discussion

A rare congenital heart defect called the aortopulmonary window (APW), which affects 0.2% to 0.6% of all congenital heart diseases, is defined by improper communication between the ascending aorta and the main pulmonary artery [3]. The two opposing conotruncal ridges that divide the truncus arteriosus into the aorta and pulmonary trunk fail to fuse, which leads to this medical condition [4].

John Elliotson gave the first description of this condition in 1830 [5], while Mori and Richardson both categorized it in 1978 and 1979, respectively [6, 7]. Mori's categorization, which divides APW into three morphological kinds, is more commonly used: type I, proximal: the rounded defect is present between the ascending aorta and the pulmonary trunk, above the semilunar valves; and type II, distal: the communication involves the pulmonary bifurcation, is situated at the level of the right pulmonary artery (RPA), and is shaped like a spiral curve. The aortopulmonary septum is completely absent in type III, a compound type that combines types I and II, as a result of a combination of proximal and distal abnormalities, simulating a common truncus arteriosus [6]. Recent studies reported that type I is the commonest type in terms of frequency, followed by type II and type III [8].

Other cardiac abnormalities, namely, ventricular and atrial septal defects, coronary artery abnormalities, or conotruncus malformations such as transposition of the great arteries and tetralogy of Fallot, as well as coarctation of the aorta and an interrupted aortic arch, can all be associated with APW [3-9], or they can be isolated as in our case.

Few cases of late-presenting APW in adults have been documented, and practically all of them presented with Eisenmenger; patients were between the ages of 18 and 60. Patients with symptoms and those who were asymptomatic up until the ages of 22, 27, and 50 have both been reported. The longest-lasting documented instance was among these reported cases, with one patient aged 46 years and another deceased at the age of 60. Additionally, two reported cases of APW in symptomatic females between the ages of 22 and 30 who hadn't experienced Eisenmenger syndrome were found in the literature search [10-11].

Research indicates that the median survival age for uncorrected APW is 33 years, the longest known survival time for untreated APW with Eisenmenger syndrome is 60 years, and our patient has impressively survived without any evidence of Eisenmenger syndrome [12-13]. The combination of a very late discovery of type 1 APW in a remarkably asymptomatic patient and the ultrasonographic diagnosis of an isolated small aortopulmonary septal defect and its restrictive nature makes our case interestingly unique in all aspects.

## Conclusions

Aortopulmonary window (APW) is a congenital heart disease that is rarely reported in the literature, with hemodynamic consequences inherently attributed to large defects. Yet, small defects with no clinical symptoms and a longer survival rate into adulthood do occur, albeit rarely. Hence, with an appropriately high index of suspicion, practitioners should be cognizant of this entity in order to avoid misdiagnosis or underdiagnosis and to improve follow-up.
